# Bazedoxifene, a Postmenopausal Drug, Acts as an Antimalarial and Inhibits Hemozoin Formation

**DOI:** 10.1128/spectrum.02781-21

**Published:** 2022-05-26

**Authors:** Renu Sudhakar, Navin Adhikari, Saniya Pamnani, Abhipsa Panda, Manish Bhattacharjee, Zeba Rizvi, Sadaf Shehzad, Dinesh Gupta, Puran Singh Sijwali

**Affiliations:** a CSIR-Centre for Cellular and Molecular Biology, Hyderabad, Telangana, India; b International Centre for Genetic Engineering and Biotechnology, New Delhi, Delhi, India; c Academy of Scientific and Innovative Research, Ghaziabad, Uttar Pradesh, India; University of Illinois at Urbana Champaign

**Keywords:** selective estrogen receptor modulator, SERM, tamoxifen, raloxifene, malaria, *Plasmodium*, drug repurposing, hemozoin

## Abstract

Despite a remarkable improvement in health care and continued drug discovery efforts, malaria control efforts are continuously challenged by the emergence of drug-resistant parasite strains. Given a long and risky development path of new drugs, repurposing existing drugs for the treatment of malaria is an attractive and shorter path. Tamoxifen, a selective estrogen receptor modulator (SERM) for the treatment and prevention of estrogen receptor-positive breast cancer, possesses antibacterial, antifungal, and antiparasitic activities. Hence, we assessed tamoxifen, raloxifene, and bazedoxifene, which represent the first-, second-, and third-generation SERMs, respectively, for antimalarial activity. Raloxifene and bazedoxifene inhibited the erythrocytic development of Plasmodium falciparum with submicromolar 50% inhibitory concentration (IC_50_) values. Among the three, bazedoxifene was the most potent and also decreased P. berghei infection in female mice but not in male mice. However, bazedoxifene similarly inhibited P. falciparum growth in erythrocytes of male and female origin, which highlights the importance of sex-specific host physiology in drug efficacy. Bazedoxifene was most potent on early ring-stage parasites, and about 35% of the treated parasites did not contain hemozoin in the food vacuole. Bazedoxifene-treated parasites had almost 34% less hemozoin content than the control parasites. However, both control and bazedoxifene-treated parasites had similar hemoglobin levels, suggesting that bazedoxifene inhibits hemozoin formation and that toxicity due to accumulation of free heme could be a mechanism of its antimalarial activity. Because bazedoxifene is in clinical use and bazedoxifene-chloroquine combination shows an additive antiparasitic effect, bazedoxifene could be an adjunctive partner of currently used antimalarial regimens.

**IMPORTANCE** The emergence and spread of drug-resistant strains of the human malaria parasite Plasmodium falciparum has necessitated new drugs. Selective estrogen receptor modulators are in clinical use for the prevention and treatment of breast cancer and postmenopausal osteoporosis. We demonstrate that bazedoxifene, a third-generation selective estrogen receptor modulator, has potent inhibitory activity against both susceptible and drug-resistant strains of Plasmodium falciparum. It also blocked the development of Plasmodium berghei in mice. The inhibitory effect was strongest on the ring stage and resulted in the inhibition of hemozoin formation, which could be the major mechanism of bazedoxifene action. Hemozoin is a nontoxic polymer of heme, which is a by-product of hemoglobin degradation by the malaria parasite during its development within the erythrocyte. Because bazedoxifene is already in clinical use for the treatment of postmenopausal osteoporosis, our findings support repurposing of bazedoxifene as an antimalarial.

## INTRODUCTION

Malaria is a parasitic disease of global importance owing to its worldwide distribution, large number of cases and deaths every year, resistance to the majority of commonly used antimalarials, and unavailability of a broadly effective vaccine. It is caused by the protozoans of the genus *Plasmodium* and affects several vertebrates, including humans. P. falciparum and P. vivax are responsible for the majority of human malaria cases, with P. falciparum being the most fatal. The parasite has a complex life cycle that completes in two hosts. *Anopheles* mosquitoes transmit the infection and support sexual development, and humans suffer from the disease and support asexual development. Both preventative measures (insecticide-treated bed nets and indoor residual spraying) and artemisinin-based combination therapies have decreased the burden of malaria in recent years (1). The recommendation of WHO to use malaria vaccine RTS,S in children at high risk of malaria in certain countries is very encouraging and will likely decrease malaria burden. However, the emergence of resistance to artemisinins and gradual loss of the efficacy of preventative measures necessitate new antimalarials, improved preventative measures, and treatment modalities. Although there are 13 antimalarial agents in clinical development at present, some of these might not reach their use in humans due to several risks associated with the drug discovery path (2). Moreover, the traditional drug discovery path is slow, with no new drugs expected for a number of years. Hence, assessment of the antimalarial activity of drugs for other diseases is an attractive and shorter path toward the identification of new antimalarials.

Selective estrogen receptor (ER) modulators (SERMs) are potential antimalarial drugs (3), which include tamoxifen, raloxifene, and bazedoxifene. These compounds modulate ER activity in a tissue-dependent manner. Tamoxifen is the first-generation SERM developed in the 1970s. It exerts ER antagonist activity in breast cancer patients and has been prescribed for the treatment of ER-positive breast cancer as well as for prevention of breast cancer in high-risk populations. However, it stimulated ER activity in bone and endometrium tissues and was found to be associated with increased risk of endometrial cancer (4, 5). The second-generation SERM raloxifene showed increased bone mineral density and lower risk of invasive breast cancer in clinical trials than tamoxifen. In contrast to tamoxifen, raloxifene did not appear to be associated with the risk of endometrium cancer, and the U.S. Food and Drug Administration (FDA) has approved it for the treatment of osteoporosis (6, 7). The third-generation SERM bazedoxifene also increased bone mineral density and reduced the incidence of vertebral fracture in clinical trials in postmenopausal women, and it is in use for the treatment of postmenopausal osteoporosis in the United States, Japan and in the European Union (8). Bazedoxifene has also been shown to have an inhibitory effect on colon cancer (9), triple-negative breast cancer (10), pancreatic cancer, and gastric cancer (11). The anticancer effects of bazedoxifene are attributed to the inhibition of the interleukin-6/glycoprotein 130/signal transducer and activator of transcription 3 (IL-6/GP130/STAT3) signaling pathway (10).

Some SERMs (tamoxifen, toremifene, 4-hydroxytamoxifen and clomiphene) have been shown to have inhibitory activity against a wide range of human pathogens, including fungi (12–14), viruses (3, 15–17) and bacteria (18–20). Tamoxifen has been reported to target the cell membrane and other intracellular molecules in some bacteria, which could be the mechanisms of its antibacterial activity (18, 20).

Tamoxifen showed antileishmanial activity in *in vitro* culture and increased the survival of infected animals (21–25). The growth of Toxoplasma gondii in *in vitro* culture and mouse models was inhibited by tamoxifen, which has been attributed to antiestrogen activity or induction of xenophagy by tamoxifen in infected cells (26, 27). Tamoxifen treatment also reduced parasite load in mice and hamsters infected with *Taenia crassiceps* and Taenia solium, respectively (28, 29). Stimulation of host cytokine response and direct effects of tamoxifen on *T. crassiceps* and *T. solium* have been attributed to this antiparasitic effect. Notably, the protective response to *T. crassiceps* was significantly higher in female (80%) than in male (50%) mice (28), suggesting a role of sex-specific host physiology in the inhibitory activity of tamoxifen. Tamoxifen blocked the growth of Trypanosoma cruzi in *in vitro* culture at micromolar concentrations but did not exhibit any effect in a mouse model even at a dose of 50 mg/kg/day for 15 to 20 days (30).

Tamoxifen, clomiphene, and tamoxifen analogs have been shown to block the asexual erythrocytic development of P. falciparum and liver-stage development of P. berghei (31–34). In a recent study, *in vitro* treatment of P. falciparum with tamoxifen and 4-hydroxytamoxifen for 96 h resulted in the inhibition of asexual development with 50% inhibitory concentration (IC_50_) values of 16.7 μM and 5.8 μM, respectively (35). In the same study, tamoxifen treatment of P. berghei-infected mice decreased parasitemia by 1.6- to 2.7-fold and inhibited cerebral malaria-like symptoms compared with the untreated P. berghei-infected mice. However, the treatment (40 mg/kg/day) was started 1 week before the infection and continued during the entire course of infection, which was quite long compared with that recommended for an antimalarial therapy (35).

Because tamoxifen has antimalarial activity, we assessed the newer-generation SERMs raloxifene and bazedoxifene for antimalarial activity.

## RESULTS AND DISCUSSION

### Bazedoxifene and raloxifene show antimalarial activity.

Tamoxifen, raloxifene, and bazedoxifene represent the first-, second-, and third-generation SERMs, respectively. We assessed the three SERMs for effects on the asexual erythrocytic-stage development of P. falciparum 3D7 and Dd2 strains. The 3D7 strain is susceptible to all the commonly used antimalarials, including chloroquine and pyrimethamine, whereas Dd2 is resistant to pyrimethamine. Bazedoxifene showed the most potent antimalarial effect on both 3D7 and Dd2, which was over 22-times more than that of tamoxifen (Table 1). A previous study assessed the antimalarial activity of tamoxifen on asexual erythrocytic development of P. falciparum over a 96-h treatment period and showed inhibition with an IC_50_ of 16.7 μM (35). The IC_50_ values of tamoxifen for the 48-h treatment period in our study (3.6 μM) is significantly lower than the reported value. We do not have an explanation for such a difference in our and the reported IC_50_ values.

**TABLE 1:** Antimalarial activity of SERMs

SERM^*a*^	IC_50_ (μM)^*b*^	
	3D7	Dd2
Bazedoxifene	0.160 ± 0.003	0.159 ± 0.003
Raloxifene	0.761 ± 0.024	0.576 ± 0.008
Tamoxifen	3.611 ± 0.211	3.371 ± 0.390
Chloroquine	0.018 ± 0.002	0.042 ± 0.001
Pyrimethamine	0.015 ± 0.0002	26.760 ± 2.99

a Compounds were assessed for inhibition of the asexual erythrocytic stage development of P. falciparum strains.

b The IC_50_ values are the means of at least three independent experiments with SD, each performed in duplicate.

### *In vivo*, but not *in vitro*, antimalarial activity of bazedoxifene is influenced by the host sex.

**FIG 1:**
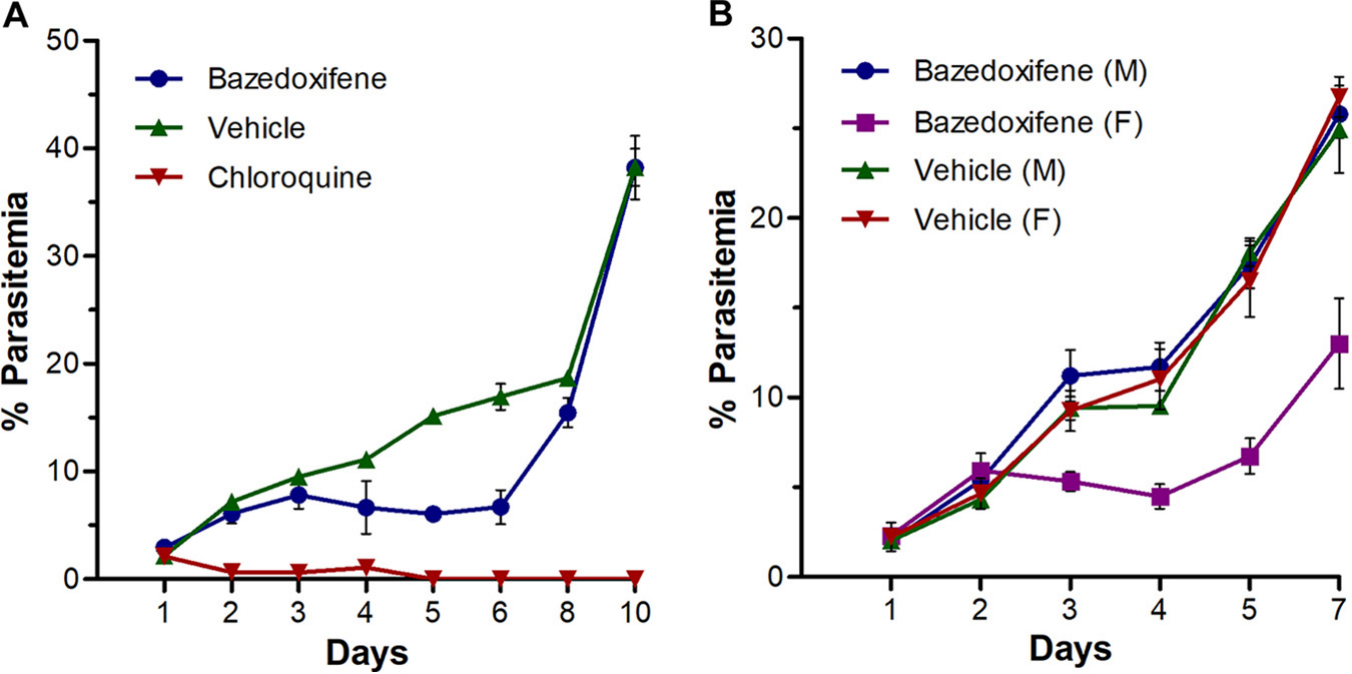
Effect of bazedoxifene on P. berghei ANKA-infected mice. BALB/c mice were infected with P. berghei ANKA and treated with bazedoxifene, vehicle, or chloroquine on days 1 to 4. The progress of infection was monitored by observing Giemsa-stained blood smears of mice and is shown as percent parasitemia over days post-treatment. (A) Only female mice were used, and treatment (5 mg/kg/day) was given through the oral route twice a day. (B) Both male (M) and female (F) mice were used, and treatment (10 mg/kg/once a day) was given intraperitoneally.

As bazedoxifene has the strongest antimalarial effect on P. falciparum, we next assessed it for antimalarial activity in P. berghei ANKA-infected mice. Compared to the vehicle control, bazedoxifene decreased parasite growth in female mice whether administered orally or intraperitoneally (Fig. 1A and B). However, it did not show any inhibitory effect in male mice (Fig. 1B), suggesting that bazedoxifene has sex-dependent antimalarial activity under *in vivo* conditions. While the *in vivo* antimalarial activity of bazedoxifene at the concentration used was modest on P. berghei ANKA-infected female mice, all three SERMs were similarly potent on P. falciparum 3D7 cultured in red blood cells (RBCs) from males and females (Table 2), which suggests that the sex-dependent antimalarial activity of bazedoxifene is not associated with the inherent nature of RBCs from different sexes. Instead, it could be due to sex-specific differences in the host physiology. Previous studies have also shown association of sex differences with morbidity of P. chabaudi-infected BALB/c mice in which treatment of mice with estrogen reduced disease symptoms in female mice (36, 37). Tamoxifen treatment has also been shown to decrease *Taenia crassiceps* load more in female than in male mice (28). Hence, it appears that the presence of physiological levels of estrogen adjuvanted the effect of bazedoxifene in female mice in our study. We could not assess the antimalarial effect of bazedoxifene at higher doses (>10 mg/kg) due to the insolubility in the vehicle, and it may show stronger antimalarial activity in both male and female mice at higher doses. To test if estrogen has antimalarial activity, the P. falciparum 3D7 strain was cultured in the presence or absence of 17β-estradiol using RBCs from male or females. We did not observe complete inhibition of parasite growth even at the highest concentration used (45.9 mM), and 50 to 60% growth inhibition was observed at concentrations of 11.5 to 45.9 mM. As the IC_50_ value of bazedoxifene is several thousand-fold lower than the 17β-estradiol concentration required for comparable inhibition, it is likely that the mode of action of bazedoxifene is different from that of 17β-estradiol. It is possible that bazedoxifene targets a parasite pathway.

**TABLE 2:** Antimalarial activity of SERMs on P. falciparum cultured in RBCs from males and females

SERM^*a*^	IC_50_ (μM)^*b*^	
	RBCs (male)	RBCs (female)
Bazedoxifene	0.141 ± 0.016	0.160 ± 0.001
Raloxifene	0.797 ± 0.005	0.959 ± 0.136
Tamoxifen	4.280 ± 0.508	4.654 ± 0.362

a Compounds were assessed for inhibition of the asexual erythrocytic-stage development of P. falciparum 3D7 strain using RBCs from males and females.

b The IC_50_ values are the means of at least three independent experiments with SD, each performed in duplicate.

### Bazedoxifene affects ring stage the most and inhibits hemozoin formation.

The three SERMs tested here have been shown to bind estrogen receptor-α, and human erythrocytes of both males and females contain estrogen receptor-α as well as estrogen receptor-β (38). Several off-targets of SERMs have been reported to be associated with their diverse activities, such as increased membrane permeability in some bacteria (18, 20), inhibition of calmodulin in mammalian cells and Saccharomyces cerevisiae, inhibition of protein kinase C (PKC) in HIV-infected cells, and inhibition of IL-6/IL-6R/STAT3 signaling in triple-negative breast cancer cell lines (3, 12, 39–41).

**FIG 2:**
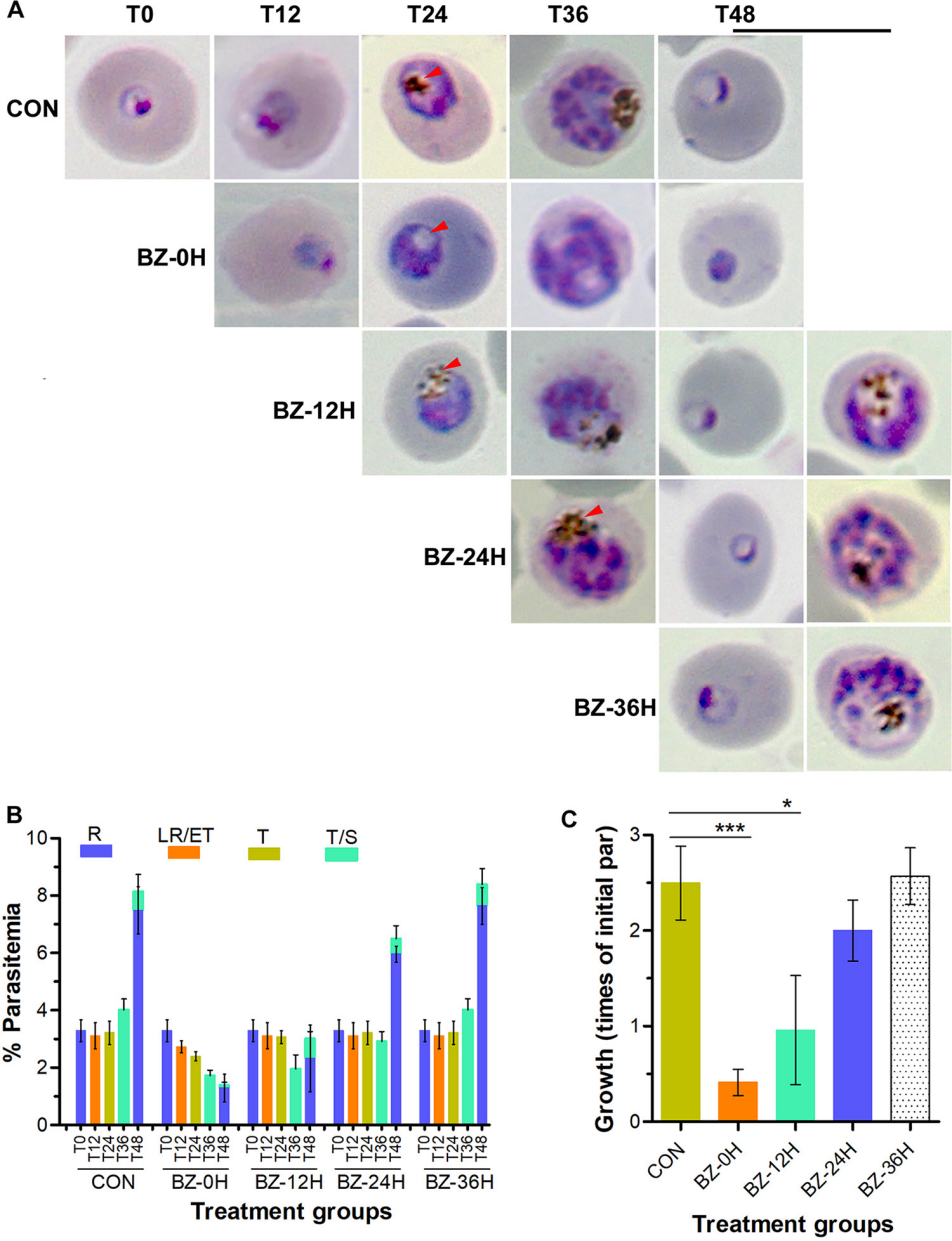
Stage-dependent effect of bazedoxifene. Synchronized P. falciparum 3D7 parasites were treated with DMSO at 0 h (CON) or bazedoxifene at 0 (BZ-0H), 12 (BZ-12H), 24 (BZ-24H), and 36 (BZ-36H) h of post-synchronization and grown until the end of the 48-h development cycle. Smears were made at 0 (T0), 12 (T12), 24 (T24), 36 (T36), and 48 (T48) h during the development cycle and observed for parasite morphology and parasitemia. (A) The images of Giemsa-stained parasites show parasite maturation with time. The arrowhead indicates the food vacuole, which contains hemozoin (brownish substance) in trophozoites (T24 and T36) of all the treated groups but not that of the BZ-0H group. (B) The graph shows percentage (% parasitemia) of different parasite stages (rings, R; late rings/early trophozoites, LR/ET; trophozoites, T; trophozoites/schizonts, T/S) in different treatment groups at the indicated time points of the development cycle. (C) The graph shows parasite growth as times of the initial parasitemia (initial par) at the end of 48-h development cycle in different treatment groups. Data in B and C represent the mean of three independent experiments, with standard deviation (SD) shown as error bars. Statistical significance is indicated for data sets showing significant *P* values only (*, *P* < 0.05 to 0.01; ***, *P* < 0.001).

To gain insights into the mechanism of the antimalarial activity of bazedoxifene, we evaluated bazedoxifene-treated parasites for morphology and maturation. Synchronized P. falciparum 3D7 parasites were treated with dimethyl sulfoxide (DMSO) at early ring stage (0 h) or bazedoxifene at early ring (0 h), late ring (12 h), trophozoite (24 h), and late trophozoite/schizont (36 h) stages of development. The parasites were allowed to grow until the end of the development cycle (48 h). Giemsa-stained smears were made and evaluated for the effect on parasite morphology and maturation. Treatment at the early ring (0 h) and late ring (12 h) stages had a stronger inhibitory effect than treatment at the trophozoite stage (24 h), whereas treatment at the late trophozoite/schizont stages (36 h) did not have any effect (Fig. 2). Parasites treated at the trophozoite (24 h) and late trophozoite/schizont stages (36 h) matured normally and produced new rings almost like the control group, indicating that bazedoxifene at the concentration used does not affect egress and invasion of erythrocytes by merozoites (Fig. 2). The stronger inhibitory effect of bazedoxifene treatment at early ring stage could be due to longer treatment duration (48 h) than the treatments at trophozoite (24-h treatment duration) and late trophozoite/schizont stages (12-h treatment duration). It is also possible that inhibition of hemozoin formation at the ring stage results in a stronger inhibitory effect than that at later parasite stages. The antifungal activity of some SERMs has been shown to be through calmodulin inhibition (12, 41). As calmodulin inhibition primarily affects invasion of erythrocytes by merozoites (42–44) and bazedoxifene had no effect on invasion of erythrocytes by merozoites, calmodulin inhibition is unlikely to be the mechanism of the antimalarial activity of bazedoxifene. Notably, a significant number of trophozoites developed from the parasites treated at early ring stage (0 h) did not contain hemozoin in the food vacuole (Fig. 2A), suggesting that bazedoxifene interferes with hemoglobin degradation and/or hemozoin formation.

**FIG 3:**
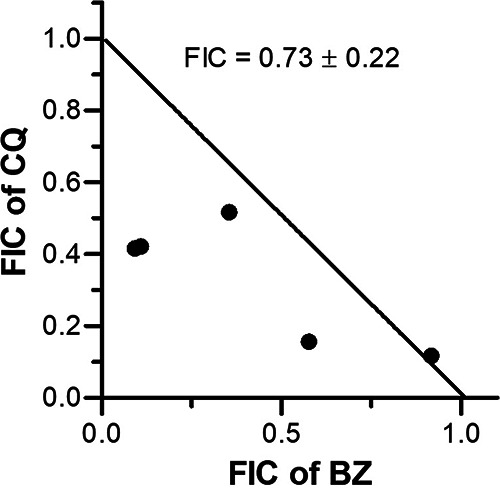
Antiparasitic effect of the bazedoxifene-chloroquine combination. Synchronized P. falciparum 3D7 parasites were cultured in the presence of DMSO or varied concentrations of bazedoxifene-chloroquine combinations or chloroquine (CQ) or bazedoxifene (BZ) for one development cycle. The 50% inhibitory concentration of each compound alone and in combination was determined to calculate fractional inhibitory concentrations (FICs) and construct the isobologram. The isobologram indicates additive interaction for the bazedoxifene-chloroquine combination (ΣFIC of 0.5 to 1.0, <0.5, and >1.5 are indicative of additive, synergistic, and antagonistic interactions, respectively).

Hemozoin is a by-product of hemoglobin degradation by the malaria parasite during its development in RBCs. Multiple parasite proteases, including the cysteine proteases falcipains and aspartic proteases plasmepsins, have been shown to degrade hemoglobin in the food vacuole, a lysosome-equivalent organelle, for obtaining amino acids and maintaining osmotic stability of the infected RBC (45–52). This process also releases heme that would be toxic for the parasite; hence, the parasite converts free heme into hemozoin, which is an inert blackish biocrystal in the food vacuole (53, 54). Antimalarials belonging to the 4-aminoquinoline group, like chloroquine, inhibit hemozoin formation, resulting in the death of the parasite due to accumulation of free heme/heme-chloroquine complex (55, 56). Importantly, a bazedoxifene-chloroquine combination showed an additive antiparasitic effect (Fig. 3), which could be, in part, due to inhibition of hemozoin formation by both of these compounds. This supports evaluation of bazedoxifene in combination with the 4-aminoquinoline group of drugs for the treatment of malaria.

**FIG 4:**
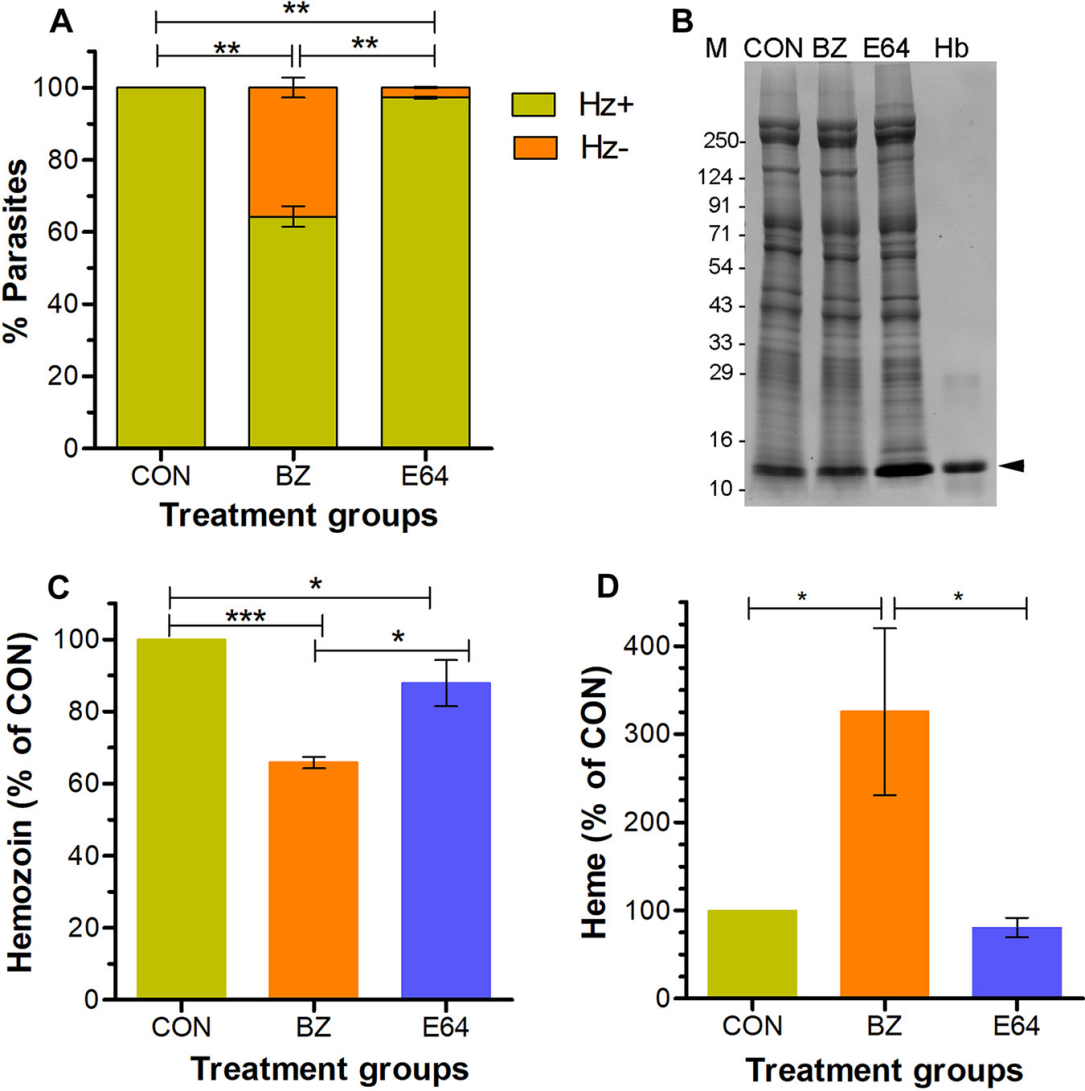
Effect of bazedoxifene treatment on hemoglobin catabolism. Synchronized P. falciparum 3D7 early ring-stage parasites were grown in the presence of bazedoxifene (BZ), E64, or DMSO (CON) for 24 h and assessed for the presence of hemozoin in the food vacuole (A), hemoglobin level (B), and hemozoin content (C). (A) The graph shows the number of parasites with (Hz+) or without (Hz−) hemozoin as a percentage of the total number of parasites. At least 500 parasites were observed for hemozoin, and the data represent the mean of three independent experiments, with SD represented by error bars. The statistical significance of the difference between the Hz− data sets is indicated. (B) Equal amounts of the total parasite lysates from the indicated treatment groups and purified hemoglobin (Hb) were resolved on a gradient SDS-PAGE gel. The Coomassie-stained SDS-PAGE gel shows protein profiles of the lysates with undegraded hemoglobin at around 14 kDa that is indicated by the arrowhead. The sizes of protein markers are in kDa (M). (C) Hemozoin was purified from the indicated treatment groups and solubilized into heme, and the amount of heme was estimated as a measure of hemozoin. The graph shows hemozoin amount in BZ and E64 treatment groups as percentage of the hemozoin amount in the control group. The data represent the mean of three independent experiments, with SD represented as error bars. (D) Free heme was isolated from the indicated treatment groups. The amount of free heme (heme) is shown as a percentage of the heme in control parasites. The data represent the mean of three independent experiments, with SD represented as error bars. Statistical significance for data sets in A, C, and D is indicated (*, *P* < 0.05 to 0.01; **, *P* < 0.01 to 0.001; ***, *P* < 0.001).

To check whether the absence of hemozoin in bazedoxifene-treated parasites is due to reduced hemoglobin uptake/degradation or failure to polymerize free heme, we treated early ring-stage parasites with DMSO, bazedoxifene, or E64 for 24 h. E64 is a cysteine protease inhibitor, which has been shown to block hemoglobin degradation, leading to accumulation of undegraded hemoglobin and relatively reduced hemozoin formation (46). The parasites were analyzed for the presence of hemozoin in the food vacuole, hemoglobin level, and hemozoin content. All of the control parasites contained hemozoin in the food vacuole, whereas about 35% of the bazedoxifene-treated parasites did not have hemozoin in the food vacuole and showed hemoglobin levels comparable with the control parasites, indicating that bazedoxifene did not inhibit hemoglobin uptake/degradation (Fig. 4A and B). The E64-treated parasites had higher hemoglobin levels than control parasites, indicating inhibition of hemoglobin degradation, as has been shown earlier (45–47, 52). Several noncysteine proteases have been shown to degrade hemoglobin, which would enable the E64-treated parasites to carry out some level of hemoglobin degradation and subsequent formation of hemozoin. This might explain why the number of parasites without hemozoin in bazedoxifene-treated parasites is significantly more than in the E64-treated group. We next isolated hemozoin, solubilized it into heme, and estimated the amount of heme as a measure of hemozoin. The heme amount was about 34% less in bazedoxifene-treated parasites and about 12% less in E64-treated parasites than in control parasites (Fig. 4C), indicating that bazedoxifene inhibits hemozoin formation. The decrease in the number of hemozoin-containing parasites is comparable with the decreased hemozoin content, which together with no effect on hemoglobin uptake/degradation indicates that bazedoxifene specifically inhibits hemozoin formation. Because bazedoxifene treatment of parasites did not have a noticeable effect on hemoglobin degradation but inhibited hemozoin formation, we estimated and compared free heme contents in bazedoxifene-treated, DMSO control, and E64-treated parasites. The bazedoxifene-treated parasites had more free heme than control and E64-treated parasites (Fig. 4D), indicating accumulation of free heme, which could be responsible for the antiparasitic effect of bazedoxifene.

**FIG 5:**
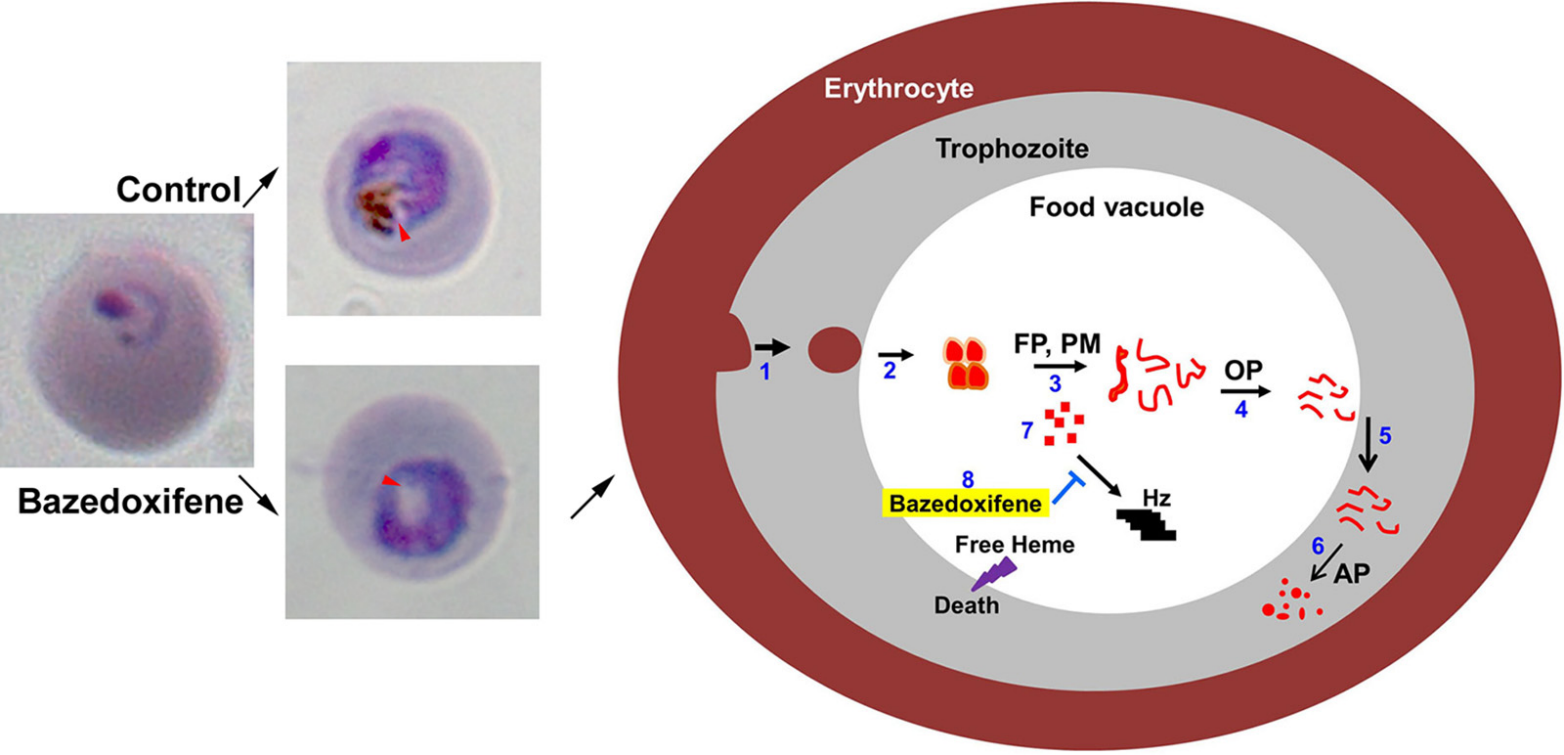
Model of bazedoxifene action. Treatment of P. falciparum at the ring stage with bazedoxifene results in trophozoites without hemozoin formation, whereas trophozoites developed from control parasites contain hemozoin in the food vacuole (marked with the arrowheads). The model summarizes hemoglobin catabolism and inhibition of hemozoin formation. The *Plasmodium* trophozoite stage developing within the erythrocyte takes up hemoglobin through a cytostome-like organelle (1) and delivers it in vesicles to the food vacuole (2). Multiple classes of proteases, including the cysteine proteases falcipains (FP) and aspartic proteases plasmepsins (PM), degrade hemoglobin (3) into oligopeptides. Oligopeptidases (OP) hydrolyze oligopeptides into smaller peptides (4), which have been proposed to be transported into the parasite cytosol (5). In the cytosol, aminopeptidases (AP) hydrolyze oligopeptides into free amino acids (6), which are used by the parasite for protein synthesis. A by-product of hemoglobin degradation is free heme (7), which is polymerized into a nontoxic polymer known as hemozoin (Hz). Bazedoxifene inhibits hemozoin formation (8), resulting in the accumulation of free heme, which could cause parasite death.

Hemozoin formation is an important detoxification mechanism in blood-sucking parasites like *Plasmodium*, helminth worms Schistosoma mansoni (57) and *Echinostoma trivolvis* (58), and the bird-infecting protozoan *Haemoproteus columbae* (59). Inability to convert heme into hemozoin is lethal for the parasite and is also the major mechanism of chloroquine action (60). The accumulation of free heme due to inhibition of hemozoin formation by bazedoxifene would damage biomolecules and membranes, leading to parasite death (Fig. 5). The hemozoin formation process is still a subject of investigation; however, HRP-II, heme detoxification protein, food vacuole proteases, and lipids have been shown to be key for heme polymerization *in vitro* (61–64). We speculate that bazedoxifene interferes in hemozoin formation by associating with lipids or proteins that are involved in this process. As chloroquine and related quinolines also cause toxicity via inhibiting heme polymerization and the bazedoxifene-chloroquine combination showed an additive antiparasitic effect, bazedoxifene should be evaluated for antimalarial activity in combination with 4-amino quinolines or any other antimalarials in use at present.

Estrogen receptors have been reported to participate in signaling events. Both ER-α and ER-β are cytosolic proteins in RBCs, which localize to the plasma membrane upon treatment with 17β-estradiol to a greater extent in RBCs from females than from males (38). The membrane-associated estrogen receptors have been shown to participate in several signaling events, including extracellular signal-regulated kinase 1/2 (ERK1/2) and AKT/phosphatidylinositol 3-kinase (PI3K) (38). Treatment of RBCs with ER agonists has been shown to decrease phosphorylation of ERK1/2 in RBCs from males and increase phosphorylation of ERK1/2 in RBCs from females. However, treatment of RBCs with ER antagonists has been shown to decrease phosphorylated AKT/PI3K levels in RBCs from both males and females. Given that the erythrocyte Raf/MEK/ERK pathway has a crucial role during parasite growth (65), it is possible that impairment of some of these signaling pathways by the SERMs used in our study could also contribute to their antimalarial activity.

This study brings up an important finding that SERMs should be evaluated for antimicrobial effects in *in vivo* disease models using both male and female animals. As bazedoxifene is already in clinical use and inhibits hemozoin formation like chloroquine, it may be used as an adjunctive treatment for malaria as a partner of 4-aminoquinolines or any currently used antimalarial regimens. New formulations allowing enhanced solubility of bazedoxifene and other SERMs will be useful for evaluation of antimalarial activity *in vivo*. Furthermore, as both chloroquine and bazedoxifene inhibit hemozoin formation, which is unique to malaria parasites, our results support development of bazedoxifene-chloroquine hybrid compounds, which will likely be potent antimalarials. It will also be interesting to study malaria incidence in postmenopausal women who are taking SERMs.

## MATERIALS AND METHODS

Parasites (P. berghei ANKA and P. falciparum 3D7 and Dd2 strains) were obtained from Malaria Research and Reference Reagent Resource Centre (MR4). Cell culture reagents were purchased from Thermo Fisher Scientific and Lonza. YOYO-1 and SYBR green 1 were from Thermo Fisher Scientific. Antimalarials (chloroquine, dihydroartemisinin, and pyrimethamine) and DMSO were from Sigma. Bazedoxifene was from Adooq Bioscience or Cayman Chemical, and tamoxifen and raloxifene were from Adooq Bioscience. All routinely used chemicals and plasticware were purchased from standard companies, such as MP Biomedicals, Sigma, Thermo Fisher Scientific, and Corning. Human blood was collected and processed according to the protocols approved by the Institutional Ethics Committee of Centre for Cellular and Molecular Biology (IEC-38-R2/2015 and IEC-38-R3/2015), and the studies reported herein abided by the declaration of Helsinki principles.

### Parasite culture and *in vitro* screening.

P. falciparum 3D7 and Dd2 strains were grown in RPMI 1640 medium with supplements (2 g/L sodium bicarbonate, 2 g/L glucose, 25 μg/mL gentamicin, 300 mg/L glutamine, 100 μM hypoxanthine, and 0.5% albumax II) and human erythrocytes at 2% hematocrit under a gas mixture (5% CO_2_, 5% O_2_, and 90% N_2_) (66). Synchronization of parasites was achieved by treatment with 5% d-sorbitol when the culture had the majority of the parasites at ring stage (67). Chloroquine was dissolved in water, and all other compounds were dissolved in DMSO to prepare 20 to 50 mM stocks.

Antimalarial activity of the test compounds was assessed by treating ring-stage parasites for one cycle as has been described earlier (45, 68, 69). The compound stocks were serially diluted 2-fold in 50 μL of culture medium across the rows of a 96-well tissue culture plate. DMSO (0.05%) or chloroquine (500 nM) were added to control wells. Fifty microliters of the parasite suspension (1 to 2% ring-infected erythrocytes at 4% hematocrit) was added to each well, and the plate was incubated in a modular incubator chamber (Billups-Rothenberg, Inc.) at 37°C for 48 to 50 h. At the end of incubation, the cells were fixed with 2% formaldehyde (in phosphate-buffered saline [PBS]) for 2 days and stained with YOYO-1 (PBS with 0.1% Triton X-100 and 10 nM YOYO-1), and the number of infected cells was determined by counting 10,000 cells using the BD Fortessa fluorescence-activated cell sorting (FACS) analyzer (*λ*_excitation_ of 491 nm and *λ*_emission_ of 509 nm). The parasitemia of chloroquine control was subtracted from that of the DMSO control and test compounds to adjust for the background. The adjusted parasitemias at different concentrations of the test compounds were normalized as percentage of the DMSO control and plotted against the concentrations of test compounds using nonlinear regression (GraphPad Prism) to determine IC_50_ values, as has been described earlier (45).

To assess antimalarial activity of tamoxifen, raloxifene, and bazedoxifene on P. falciparum cultured in RBCs from males and females, the assays were set up exactly as described above except for two modifications: parasites were maintained in RBCs from males or females and parasitemia was determined using the SYBR green 1 method (70). In brief, 100 μL of lysis buffer (20 mM Tris-Cl, 5 mM EDTA, 0.008% saponin, and 0.08% Triton X-100, pH 7.5) with SYBR green 1 (at the manufacturer’s recommended dilution) was added to each well, the plate was incubated at 37°C for 30 min, and the fluorescence of each well was measured (*λ*_excitation_ of 485 nm, *λ*_emission_ of 530 nm) using a SpectraMax iD3 multimode microplate reader. Background fluorescence was corrected by subtracting the fluorescence values of the chloroquine control from those of the DMSO control and test compounds. Fluorescence values of test compound wells were normalized as percentage of the fluorescence value of the DMSO control plotted against the concentrations of test compounds and analyzed by nonlinear regression (GraphPad Prism) to determine IC_50_ values as described earlier (45).

### Experiments in mice.

Animals were housed in a cabin-type isolator under standard environmental conditions (22 to 25°C, 40 to 70% humidity, and a 12-h/12-h dark/light photoperiod). All animal experiments were performed according to the protocol (25/2020) approved by the Institutional Animal Ethics Committees (IAEC) of the Centre for Cellular and Molecular Biology. For maintenance, one 4- to 6-week-old BALB/c mouse was infected with a frozen stock of P. berghei ANKA, and infection was monitored regularly by observing Giemsa-stained blood smear of the infected mice. Whenever required, blood was collected from the tail snip of this mouse in Alsever’s solution (2.05% glucose, 0.8% sodium citrate, 0.055% citric acid, and 0.42% sodium chloride). For treatment through the oral route, fifteen 4- to 6-week-old female BALB/c mice were infected with 1 × 10^6^ parasites intraperitoneally and were divided into three groups of five mice each; treatment was given by oral gavage once parasites appeared in the blood smear. The first group was given bazedoxifene (5 mg/kg in 200 μL PBS with 2% Tween 80 and 1% methylcellulose), the second group was given chloroquine (10 mg/kg in 200 μL PBS), and the third group was given vehicle only (200 μL of PBS with 2% Tween 80 and 1% methylcellulose). The treatments were given twice a day for 4 days. For treatment through the intraperitoneal route, eight 4- to 6-week-old female and eight male BALB/c mice were infected with 1 × 10^6^ parasites intraperitoneally, and treatment was initiated once parasites appeared in the blood. Five male and five female mice were given bazedoxifene (10 mg/kg in 100 μL of sesame oil with 10% DMSO), and three male and three female mice were given vehicle only (100 μL of sesame oil with 10% DMSO). The treatments were once a day for 4 days. Infection was monitored regularly by observing Giemsa-stained blood smears of infected mice, and parasitemia was determined by counting at least 1,000 cells and plotted over days post-treatment.

### Stage-dependent effect of bazedoxifene.

A P. falciparum 3D7 culture with a majority of ring-stage parasites was treated with sorbitol at the beginning and end of the 48-h development cycle to obtain synchronized parasites. The synchronized parasites were treated with DMSO (0.05%) at early ring stage (0 h) or bazedoxifene (480 nM, equal to 3× IC_50_ value) at early ring (0 h), late ring (12 h), trophozoite (24 h), and late trophozoite/schizont (36 h) stages of the 48-h development cycle. The cultures were allowed to grow until the end of the development cycle, and Giemsa-stained smears were prepared at 0-, 12-, 24-, 36-, and 48-h time points. The smears were observed, and images were taken using the 100× objective of a light microscope (Axio-Imager Z2). At least 2,000 cells were observed to determine the number of different parasite stages (rings, late ring/early trophozoite, trophozoite, and trophozoite/schizont) and morphologies. The parasitemia at different time points was analyzed and plotted using GraphPad Prism. A Student’s *t* test was used to compare the means of data sets.

### Hemoglobin catabolism and hemozoin formation.

Synchronized P. falciparum 3D7 parasites were treated at early ring stage with DMSO (0.03%), bazedoxifene (480 nM, equal to 3× IC_50_ value) or E64 (6.9 μM, equal to 3× IC_50_ value), grown for 24 h, and processed for making Giemsa smears and isolation of parasites. The smears were observed using the 100× objective of a light microscope. Parasites were isolated by the saponin lysis method and processed for SDS-PAGE analysis and hemozoin estimation (71). The parasite pellets were resuspended in 5× pellet volume of 10 mM Tris (pH 7.5) buffer supplemented with 2× pellet volume of 4× SDS-PAGE sample buffer (1× sample buffer contains 10% [vol/vol] glycerol, 2% [wt/vol] SDS, 0.1% bromophenol blue, 100 mM dithiothreitol [DTT], and 50 mM Tris-Cl, pH 6.8) boiled for 5 min and centrifuged at 21,500 × *g* for 30 min at room temperature. The supernatant was separated and run on a 4 to 15% gradient SDS-PAGE gel along with pure hemoglobin (Sigma, H7379) as a control, and the gel was stained with Coomassie blue to visualize proteins. The pellet was processed for hemozoin isolation as has been described previously (72). Briefly, the pellet was serially washed with 1 mL of 2% SDS (in 100 mM Na_2_CO_3_, pH 9.5), 1 mL of 2% SDS (in water), and 1 mL of Milli-Q water. The pellet was incubated with 1 mL of proteinase K solution (50 mM Tris-Cl, 100 mM NaCl, 1 mM EDTA, and 0.5% SDS, pH 7.5; proteinase K at 1 mg/mL) overnight at 60°C to digest any carryover proteins. The sample was centrifuged at 21,500 × *g* for 30 min at room temperature, the supernatant was discarded, and the pellet was washed with 1 mL of Milli-Q water. The blackish hemozoin pellet was resuspended in 1 mL of decrystallization buffer (2% SDS and 20 mM NaOH) and incubated at 37°C for 1 h to solubilize hemozoin into free heme, and absorbance was measured at 410 nm using the SpectraMax iD3 multimode plate reader. The data were analyzed and plotted using GraphPad Prism. A Student’s *t* test was used to compare the means of data sets.

### Heme estimation.

Synchronized P. falciparum 3D7 parasites were treated at early ring stage with DMSO (0.03%), bazedoxifene (480 nM, equal to 3× IC_50_ value) or E64 (6.9 μM, equal to 3× IC_50_ value) for 24 h. After treatment, parasites were isolated by the saponin lysis method and processed for estimation of free heme exactly as has been reported earlier (73). Absorption spectra of heme were recorded using the SpectraMax iD3 multimode plate reader. The heme contents of bazedoxifene-treated and E64-treated parasites were expressed as a percentage of the heme content present in the DMSO control and plotted using GraphPad Prism. A Student’s *t* test was used to compare the means of the data sets.

### Effect of the bazedoxifene-chloroquine combination on parasite development.

Stocks (400×) of bazedoxifene-chloroquine combinations in ratios of 5:1, 3:1, 1:1, 1:3, and 1:5 of their respective IC_50_ values were prepared. The stocks were serially diluted 2-fold in 50 μL of culture medium, 50 μL of parasite suspension (1% ring-infected erythrocytes at 4% hematocrit) was added to each well, cultures were grown for 48 h, and the IC_50_ values of each inhibitor in the combination were determined using the SYBR green 1 method, as has been described above and previously (70). The IC_50_ values of bazedoxifene and chloroquine alone were also determined along with their combinations. The fractional inhibitory concentration (FIC) was determined for each inhibitor in each combination (FIC = concentration of a compound that caused 50% inhibition in the combination/concentration of the compound required for 50% inhibition when used alone), as has been previously reported (68, 74). The FICs at different ratios for chloroquine-bazedoxifene combinations were plotted using linear regression analysis to construct an isobologram using GraphPad Prism. The sum of FICs (ΣFIC: FIC of bazedoxifene + FIC of chloroquine in the combination) at all combinations was used to determine mean ΣFIC, which was used to determine the nature of interaction (ΣFIC of 0.5 to 1.0, <0.5, and >1.5 are indicative of additive, synergistic, and antagonistic interactions, respectively).
